# Free Flap Donor Site Reconstruction: A Prospective Case Series Using an Optimized Polyurethane Biodegradable Temporizing Matrix

**Published:** 2015-06-26

**Authors:** Marcus J. D. Wagstaff, Bradley J. Schmitt, Yugesh Caplash, John E. Greenwood

**Affiliations:** ^a^Adult Burn Centre, Royal Adelaide Hospital, Adelaide, South Australia, Australia; ^b^Department of Plastic and Reconstructive Surgery, Royal Adelaide Hospital, Adelaide, South Australia, Australia

**Keywords:** biodegradable polyurethane, synthetic dermal matrix, dermal scaffold, free flap donor site, reconstruction

## Abstract

**Introduction:** We recently published a 10-patient case series where free flap donor site reconstruction was performed as a 2-stage procedure using an integrating biodegradable polyurethane matrix (to form a neodermis), followed by definitive closure with an autologous split-skin graft. Two issues were revealed by this pilot study that led to further modification of the biodegradable temporizing matrix. This involved alterations to the seal thickness and bonding to the foam matrix and the introduction of fenestrations to the seal. **Objective:** This article documents a second cohort of patients requiring free flap (fibular and radial forearm) donor site reconstruction with this optimized material. **Methods:** The biodegradable temporizing matrix was implanted when the free flap was detached from its donor site. Subsequent integration was monitored closely. Five weeks was the usual time of integration before delamination (seal removal), dermabrasion, and definitive closure with autograft. **Results:** Integration was complete and uncomplicated in every case, delamination occurred in 1 piece in 1 action, and subsequent graft take was 100% for every patient. Long-term scar outcomes improved compared with the pilot group. Degradation is complete by 12 months, other than occasional microscopic remnants undergoing phagocytosis. **Conclusion:** This study has reiterated that the biodegradable temporizing matrix can be implanted into humans, followed by neovascularization and integration. No infection was observed, and split-skin overgrafting was successful and uncomplicated.

We recently published a 10-patient case series where free flap donor site reconstruction was performed as a 2-stage procedure using an integrating biodegradable polyurethane matrix (to form a neodermis), followed by definitive closure with an autologous split-skin graft. On reflection, the complications and issues raised by this pilot study indicated that further modification of the Biodegradable Temporising Matrix (BTM) was necessary. First, the unfenestrated seal (bonded to the matrix and designed to prevent evaporative water loss and thus reduce wound contraction while the seal is in situ) allowed accumulation of tissue fluid in some radial forearm flap donor site reconstructions. This fluid had the potential to become infected, and did so in 2 cases. In addition, a subseal seroma prevented BTM adherence and integration in 1 patient and underlying contained wound infection in 2 patients indicated partial matrix removal. Second, when the matrix was integrated and was ready for delamination and grafting, the seal was fragile and tended to fragment during removal. This made delamination excessively time-consuming and impractical for its intended use in major burns and created the concern that bond material was perhaps being left on the surface of the integrated matrix.^1^

This prompted alterations to the seal thickness, seal bonding, and the introduction of seal fenestrations. The new pseudo-epidermal seal was 50 μm thick and bonded with a biodegradable polyurethane adhesive to the underlying 2 mm thick biodegradable polyurethane foam dermal component.

The fenestrations were introduced to allow subseal fluid to egress into the overlying dressings, preventing the collection of tissue fluid interfering with the interface between the BTM and the wound (and thereby integration) and allowing the drainage of any such reservoir that is a potential nidus for infection and preventing containment of any such infection.

Studies of this new BTM were performed in our standard porcine wound model (large White × Landrace animals, four 8 × 8 cm wounds to panniculus adiposus on the dorsum of each animal^2^^-^4). The design modifications worked as intended. All BTM seals were removed in a single action and in 1 piece. In addition, the seal bond was strong enough to tear the superficial struts of the dermal component foam, ensuring that all bond material remained on the seal *and* that the action of removal refreshed the superficial surface of the integrated foam neodermis.^5^

This article reports on the treatment course and long-term outcomes of a second cohort of 10 consecutive patients who received the new BTM.

## OBJECTIVES OF THIS STUDY

Given the modifications made to the BTM following the pilot study, the objectives were the same in this cohort, allowing comparison between the groups in the short-term utility of the new BTM (integration, delamination, and skin graft application and take) and long-term outcomes (cosmetic appearance, scar symptoms and pliability, and degree of wound contraction).

## MATERIALS AND METHODS

Following the previous pilot trial, the Human Research Ethics Committee of the Royal Adelaide Hospital proposed and endorsed the application of 3 surgeons within our Department to the Australian Therapeutic Goods Administration for use of the BTM as an unapproved Therapeutic Good under Section 41HC of the Therapeutic Goods Act 1989. This was approved at the beginning of 2014 (authorized prescriber No. 2014/013, expiry date January 28, 2015). This second program was thus not classified as a “trial.”

Ten patients were recruited and underwent harvest of a fibular osseocutaneous flap, or a radial forearm flap, for head and neck cancer. The test materials, biodegradable temporizing matrix (BTM), were manufactured, packaged, sterilized by γ-irradiation, and transported to the Royal Adelaide Hospital by PolyNovo Biomaterials Pty Ltd, Port Melbourne, Victoria.

The surgical treatment protocol was identical to the pilot study^1^; however, the BTM was hand-fenestrated by the operating surgeon prior to implantation. Measurement was also identical to the previous pilot trial, including physiotherapy-assessed outcomes.^1^ Scar appearance was assessed using 2 scar assessment scales at approximately 1 year postimplantation by the same senior physiotherapist who performed the pilot study assessments. Again, the scar assessment scales used were the Patient and Observer Scar Assessment Scale v2.0 (POSAS)^6^ and Matching Assessment using Photographs with Scars (MAPS).^7^

The POSAS consists of the Patient and Observer scales. The Patient scale consists of 6 questions (scaled 1–10, with a score of 1 representing the best scar or sensation and a score of 10 representing the worst). The Observer scale consists of 6 questions on scar characteristics (scaled 1-10, with lower scores indicating a better result).

For each scale, results range from 6 to 60. An additional question for overall opinion of the scar (score = 1–10) is included in both scales but not included in the overall score. MAPS is a scar assessment scale rating 4 scar parameters (surface, border height, thickness, and color) on a 6-point scale from −1 to 4, with pigmentation included as required. Scores may range from −5 to 17, with a score approaching 0 representing a better outcome. MAPS has been shown to have acceptable levels of intra- and interrater reliability.^7^^,^^8^

At 12 months, 3 mm punch biopsy specimens were harvested from the center of the scar in those patients surviving, and attending follow-up, to a year (*n* = 6). These were performed under local anesthetic infiltration with 2 mL of 0.25% bupivacaine and 1:400,000 adrenalin. Following processing, the slides were viewed by a senior veterinary pathologist (who has assessed polymer specimens throughout BTM development).

## RESULTS

A summary of each patient's demographics, treatment course to scar maturation, has been included (Table 1). The mean wound surface areas at the time of delamination/split-skin grafting and at 1 or more years postimplantation are recorded in Table 2 and compared with those of the pilot trial. In the current cohort, only fibular and radial forearm free flap donor sites were reconstructed. Only 2 fibular flap donor sites were included (patients 1 and 3). Neither sustained any reduction in wound surface area between donor site creation and approximately 1 year post-BTM implantation. In the 7 patients with radial forearm flap surviving until grafting, the mean wound area on the day of delamination and grafting was 99.01% that of the original wound created an average of 35 days earlier (Table 2). Once the material was delaminated and grafted, however, a degree of contraction occurred, with the last recorded mean radial forearm flap donor site scar surface area being 70.66% that of the original wounds (at ˜12 months in 4 patients).

The BTM took completely in all patients surviving until grafting and was integrating normally in patient 5, who died of an unrelated surgical complication at day 12 postimplantation. This can be seen in the third picture of each patient series (Figs 1–10).

No patient course was marred by infection (in contradistinction to the pilot group, where infection was seen in 4/10 patients).

No BTM seal delamination occurred spontaneously or prior to the scheduled removal. Mean removal occurred over the entire cohort at 36 days, dictated by the presence of tendons in the base of every wound. Surgical delamination occurred quickly, in one action and the seal was removed in one piece in all cases (see Video 1). In all cases, the superficial struts of the dermal foam component remained fixed to the bond/seal, automatically refreshing the surface of the integrated polymer neodermis.

Three patients failed to reach the 12-month time point (patients 5, 8, and 20, reaching days 12, 236, and 224, respectively), and 1 subject was lost to follow-up after day 77 (patient 4).

The remaining 6 patients had their scars assessed at approximately 1 year by the same senior physiotherapist who performed the MAPS and POSAS assessments in the pilot trial. The results are shown in Table 3. In both MAPS and POSAS, a lower scar approximates more closely to normal skin. Both assessments yielded better scores than the pilot trial (Table 3).

## DISCUSSION

### Safety

As in the pilot trial, the material was tolerated, with no overt symptom or sign of adverse reaction or hypersensitivity to its presence. No patient complained of any pain in the donor site, including during dressing changes.

### Surgical experience

BTM implantation was again straightforward, and fixation was rapid with surgical staples. Overdressing was performed using routine dressings based on routine protocols for existing dermal templates. BTM dressing changes were usually commenced on day 3, then every 3 days as indicated by the duration of action of the silver dressing (Acticoat; Smith & Nephew, Hull, United Kingdom). Splinting of the ankle for 7 days followed BTM implantation into the fibular flap donor sites and of the wrist for 5 days in the case of radial forearm flap donor sites. Once grafted, dressings with jelonet and betadine-soaked gauze every 3 days were discontinued at 14 days when graft massage and moisturization commenced.

### Integration

All BTMs integrated completely in the 9 patients who survived until grafting. The radial forearm flap group underwent delamination and was skin grafted after a mean of 35-day integration period, comparable with the pilot study (38.5 days). An integration period of 5 weeks yielded consistently successful rates of take. The fibular flap group was delaminated after a mean of 37 days as opposed to 23.33 days in the pilot study. Given our experience at this time, the authors feel that fibular and forearm donor sites may integrate over shorter time periods than 5 weeks; however, this would require further clinical evaluation.

### Delamination

The removal of the BTM seal posed a significant problem during the pilot study.^1^ Its fragility led to tearing and piecemeal removal, which, although possibly tolerable in small surgical wounds, would be completely unacceptable in its proposed indication in major deep burn injury repair. With the new seal and bond, delamination in every case was in in one action and the seal was removed in one piece in all cases (see Video 1).

### Dermabrasion

Although the quality of the superficial surface of the integrated BTM neodermis was refreshed by the tearing of the foam struts on delamination, dermabrasion was still performed to demonstrate the vascularity of the new tissue (see Video 2). The video illustrates that this dermabrasion is exceptionally light and that no polymer foam is dislodged or abraded away during this process.

### Skin grafting

Fenestrated sheet graft was applied in every case, predominantly for the enhanced cosmetic outcome possible in such small wounds. A suitable initial dressing comprised paraffin gauze, a layer of betadine-soaked gauze, and crepe bandage and usually remained until 4 or 5 days postgraft application. Graft take was 100% in the 9 case patients who survived to graft application.

### Donor site variability

**Figure 11:**
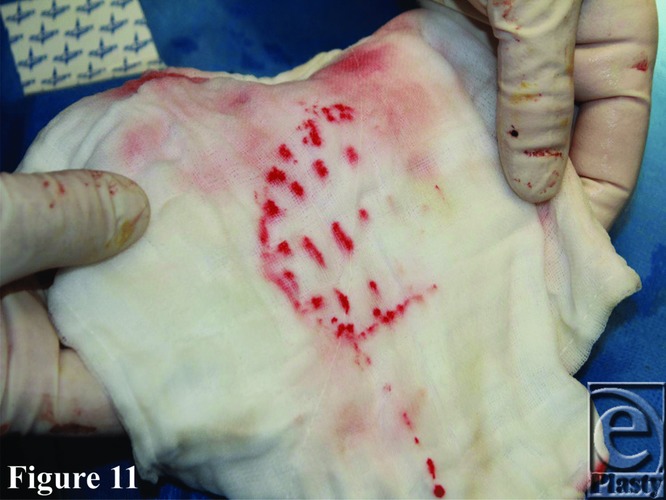
Escape of hemo-sanguinous fluid through the BTM seal fenestrations. BTM indicates biodegradable temporizing matrix.

In comparison with the pilot study, the donor sites left by the harvest of radial forearm and fibular flaps behaved identically. Both had exposed paratenon in the wound bed. The seal fenestrations prevented the collection of fluid, which could clearly be seen escaping (Fig 11).

### Infection

No signs or symptoms of infection were reported or observed in any case at any time point.

### Wound area

The fibular flap donor site repairs behaved very similarly to the pilot trial. A degree of “expansion” of wound area occurred within a few days of BTM implantation, which subsequently reversed. By 1 year postimplantation, the wound areas were almost the same as when the wounds were created (patient 1: day 0 = 59.4 cm^2^, day 367 = 60.8 cm^2^; patient 3: day 0 = 28.1 cm^2^, day 391 = 29.1 cm^2^). The radial forearm flap reconstructions demonstrated almost no contraction in the wound surface area during the integration phase, whereas the BTM seal was in situ (mean wound area as a percentage of original = 99.01%). Delamination and grafting resulted in a mean scar area of 70.66% of the original wound when persisting for 1 year or longer. This was also observed in the pilot trial, although the degree of contraction was much higher in the pilot cases where early delamination for infection was performed. Comparing the second forearm cohort against the first, both up to delamination and from delamination to 1 or more years postimplantation, there was less mean contraction resulting in a proportionally larger wound area (70.66% of the original wound size, cf 36.64% in the first cohort; Table 2). Early delamination, we believe, contributed to early and continued wound contraction due to increased myofibroblast differentiation as a result of early evaporative water loss, stimulating over-granulation through the full thickness of the BTM.

### Scarring outcomes

General improvement in scar scores was evident for this second cohort of subjects, with a greatest MAPS score of 2 and a mean MAPS score of 1.33, lower than the pilot cohort (Table 3), suggesting that the second cohort had superior cosmetic outcomes (see Video 3). Similarly, POSAS Observer scale scores all trended lower in the second cohort, again supporting more aesthetic scars. POSAS Patient scale scores were slightly higher for the second cohort with respect to total score and average score per question, although the subjects’ overall opinion score of their scar quality was superior to the pilot cohort. No subjects in the second cohort reported any symptoms of itch or pain in their scar in the 4 weeks before completing the POSAS Patient Scale, findings consistent with the pilot cohort.

### Histology (degradation)

**Figure 12:**
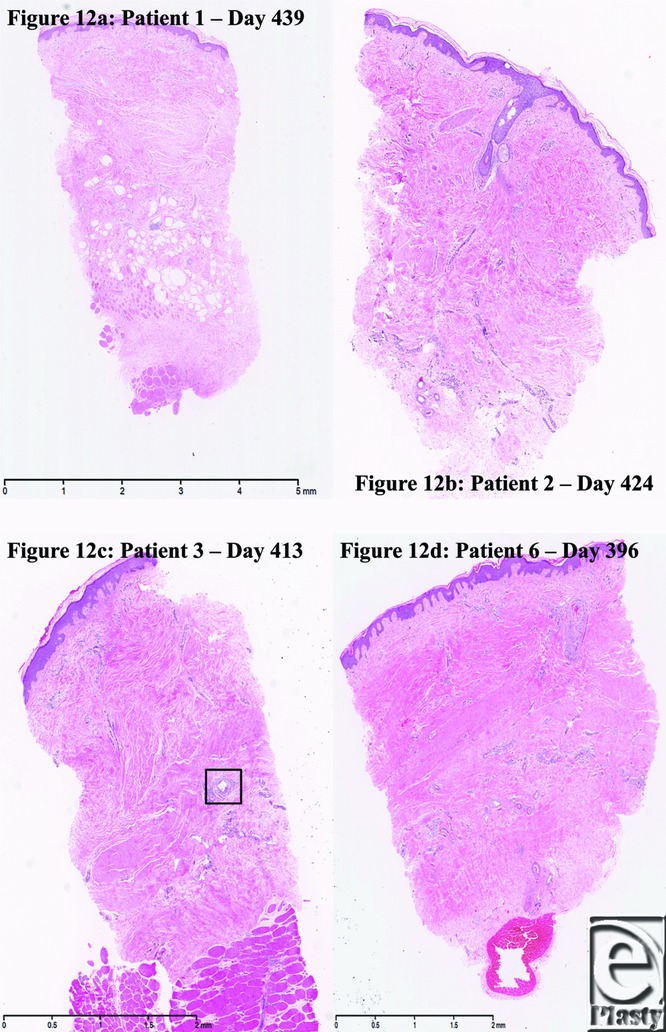

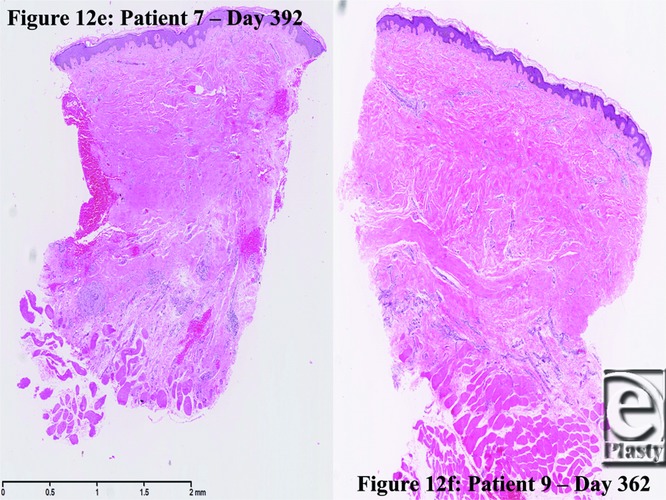
Histological appearance of scar at (a) day 439 (patient 1); (b) day 424 (patient 2); (c) day 413 (patient 3)—box surrounds polymer remnant; (d) day 396 (patient 6); (e) day 392 (patient 7); and (f) day 362 (patient 9).

**Figure 13:**
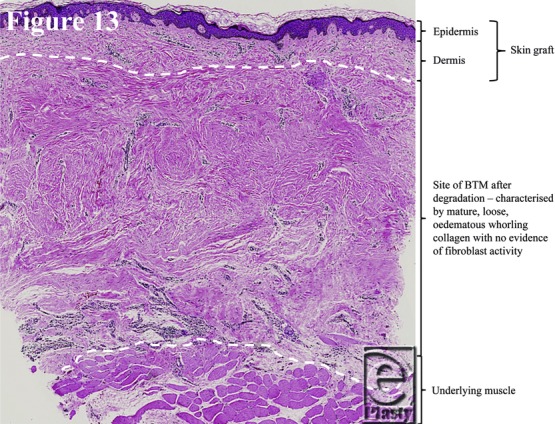
Explanatory diagram demonstrating the strata of the punch biopsy specimens.

**Figure 14:**
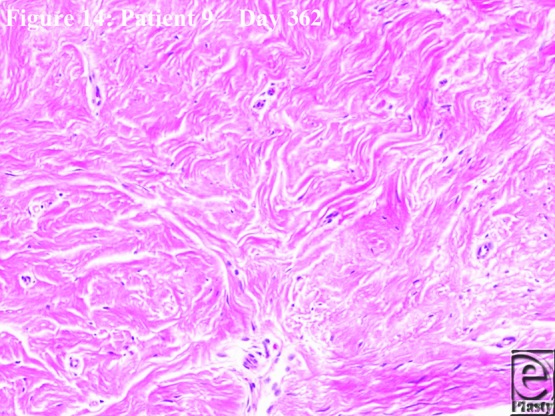
Higher magnification histological appearance of scar at day 362 (patient 9) demonstrating the mature but loose edematous whorls of collagen laid down inside the BTM and persisting after degradation. BTM indicates biodegradable temporizing matrix.

The histology slides (Figs 11–14) were produced from punch biopsy specimens taken from the center of each scar of the patients surviving to 1 year (*n* = 6). In the pilot study, microscopic remnants of polymer, surrounded by giant cells, were detectable at 12 months.^1^ The inference was that hydrolysis had been completed and residual, nonhydrolyzable fragments were phagocytosed. Biopsy specimens were taken at approximately 1 year in the current cohort. Each biopsy specimen was sectioned vertically, yielding between 3 and 5 vertical sections. Microfragments were observed, surrounded by giant cells, in 7 of 21 sections. Their histological appearance is illustrated in Figure 12*a* (from patient 3), where the largest microscopic remnant of any of the 6 subjects can be seen, and has been boxed on the figure. This series reflects the desired properties of the BTM, i.e. that the structure would be maintained until tissue integration, and that the product would largely degrade over 12 months. Figure 13 shows a punch biopsy specimen stratified and Figure 14, a high magnification of the whorling collagen characteristic of the intrafoam deposition, which persists after the polymer has degraded. An important feature of all of the punch biopsy specimens is the lack of fibroblast infiltration in the BTM collagen, indicating that, at 12 months, this collagen is mature.

The changes made to the BTM as a result of observations made during the pilot trial have now been assessed in a porcine model^5^ and a human free flap cohort. The BTM now functions according to its design parameters. These alterations have optimized the material and given us the foundation for a 5-patient significant burn trial (20%–50% total body surface area full-thickness burns). There is no clinical evidence of hypersensitivity or other pathological reaction to the integrating polyurethane component, allowing safe implantation into clean, debrided human wounds after appropriate hemostasis. It integrates by neovascularization. Once integrated and delaminated, it sustains split-skin graft take. While the pilot study demonstrated BTM's tolerance to infection and ability to continue to support vascularization in the presence of treated infection, the current study illustrates that infective problems can be avoided. The long-term results (in terms of scar pliability, appearance, and patient/therapist opinion) are superior to the pilot material (Table 3). This supports the necessity of the changes, which we feel were important in the subsequent lack of complications.

## CONCLUSIONS

This second cohort received an improved, and optimized, version of the BTM. No safety issues or infections resulted, delamination was easy and rapid, and graft take was complete in all cases with improved long-term outcomes. Degradation is virtually complete by 12 months, other than occasional microscopic remnants undergoing phagocytosis.

## Figures and Tables

**Figure 1:**
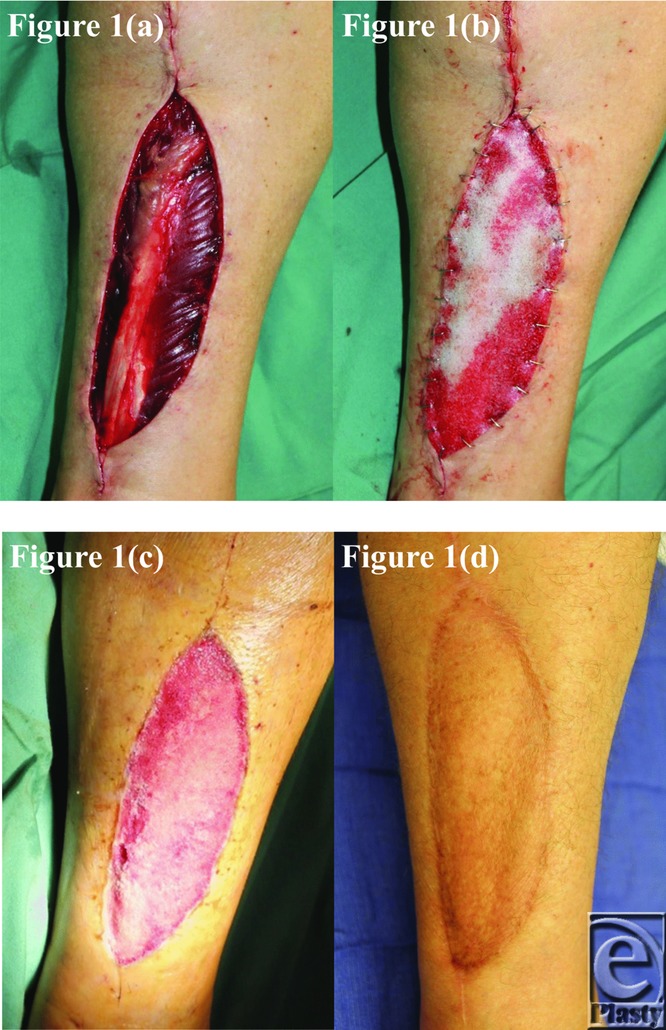
Patient 1 temporal series: (a) day 0 wound; (b) day 0 BTM implanted; (c) day 34 BTM delaminated and dermabraded; and (d) day 367 final graft BTM/scar. BTM indicates biodegradable temporizing matrix.

**Figure 2:**
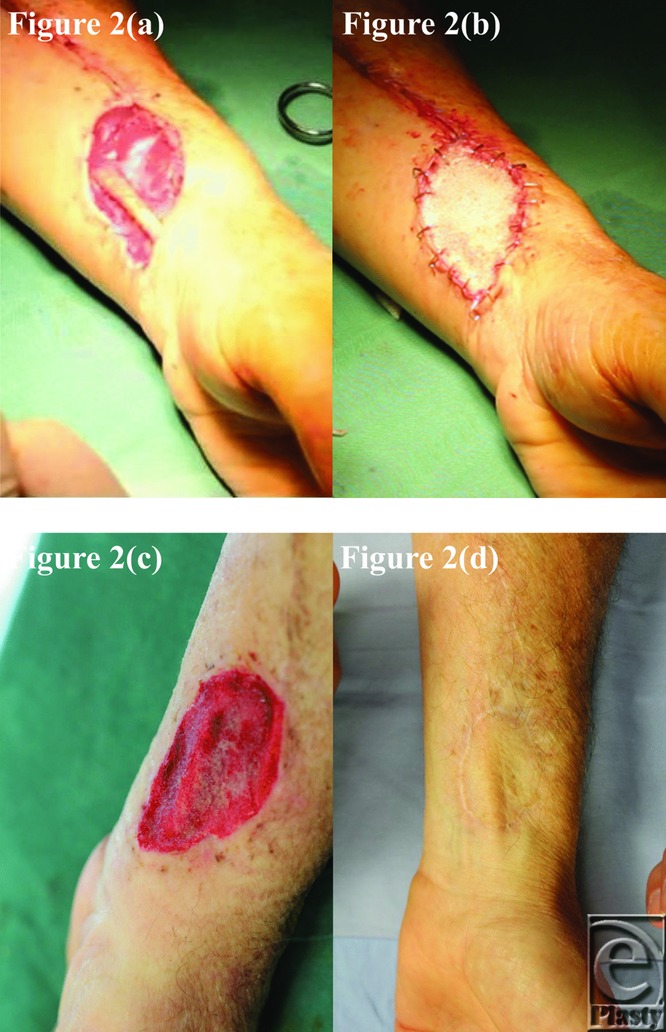
Patient 2 temporal series: (a) day 0 wound; (b) day 0 BTM implanted; (c) day 34 BTM delaminated and dermabraded; and (d) day 424 final graft BTM/scar. BTM indicates biodegradable temporizing matrix.

**Figure 3:**
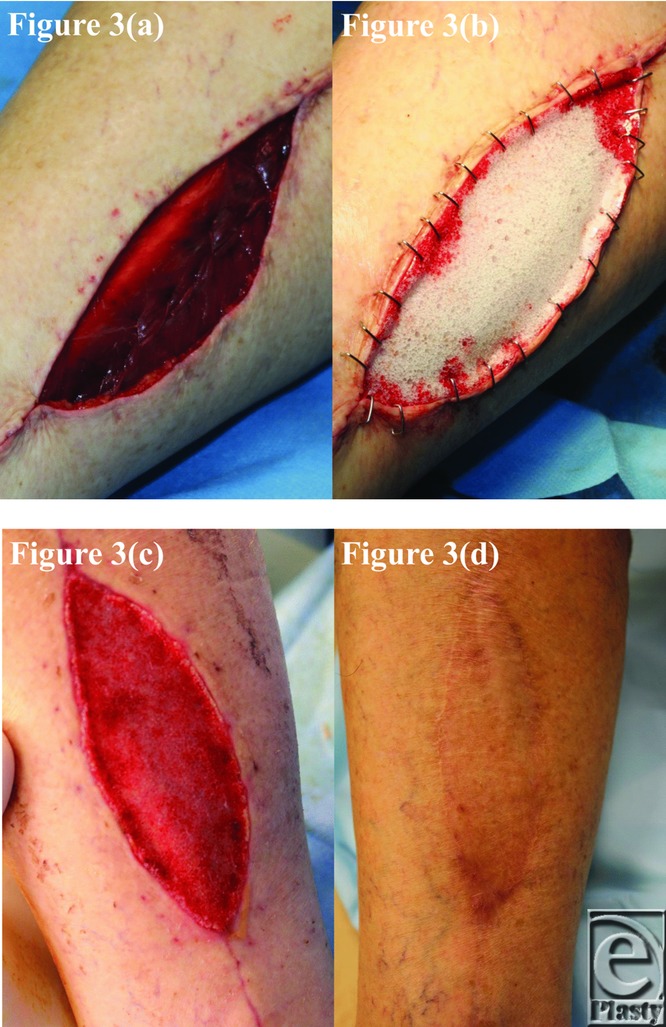
Patient 3 temporal series: (a) day 0 wound; (b) day 0 BTM implanted; (c) day 40 BTM delaminated and dermabraded; and (d) day 391 final graft BTM/scar. BTM indicates biodegradable temporizing matrix.

**Figure 4:**
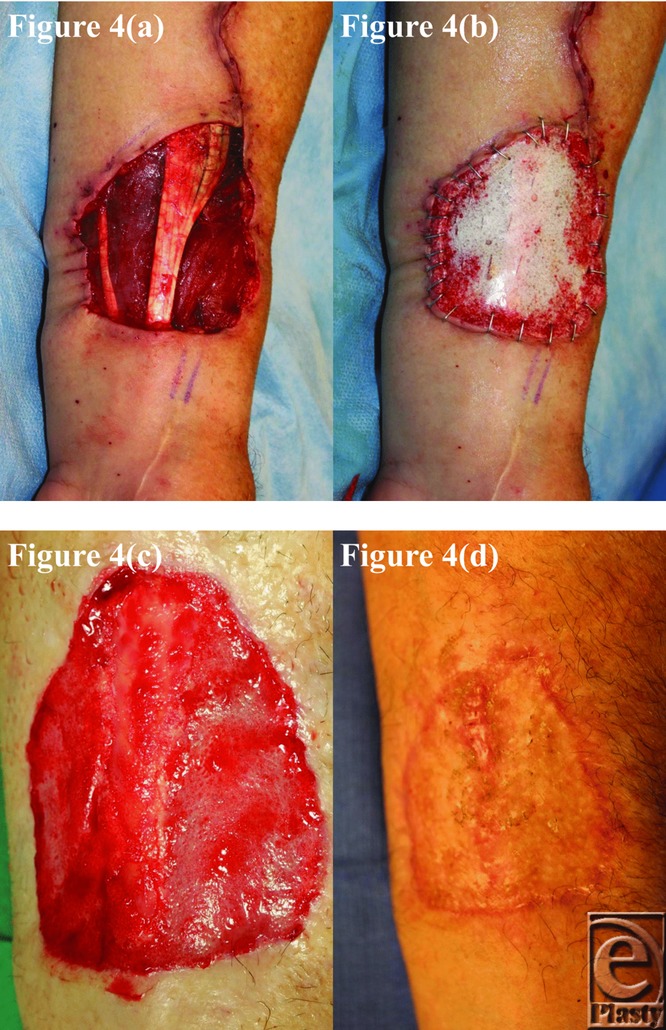
Patient 4 temporal series: (a) day 0 wound; (b) day 0 BTM implanted; (c) day 43 BTM delaminated and dermabraded; and (d) day 77 final graft BTM/scar. BTM indicates biodegradable temporizing matrix.

**Figure 5:**
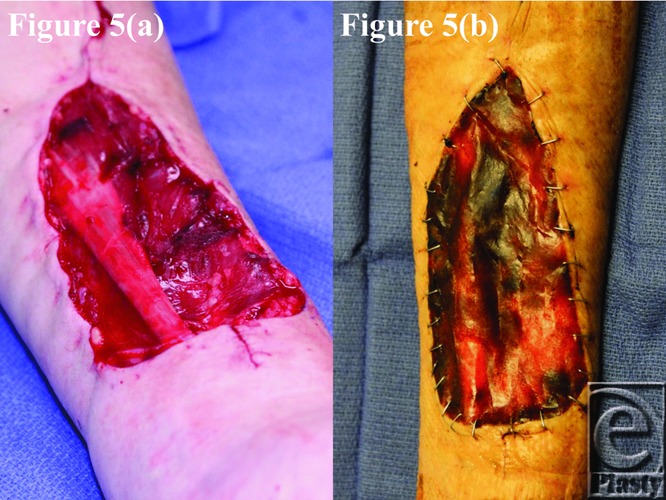
Patient 5 temporal series: (a) day 0 wound; (b) day 11 BTM progress. BTM indicates biodegradable temporizing matrix.

**Figure 6:**
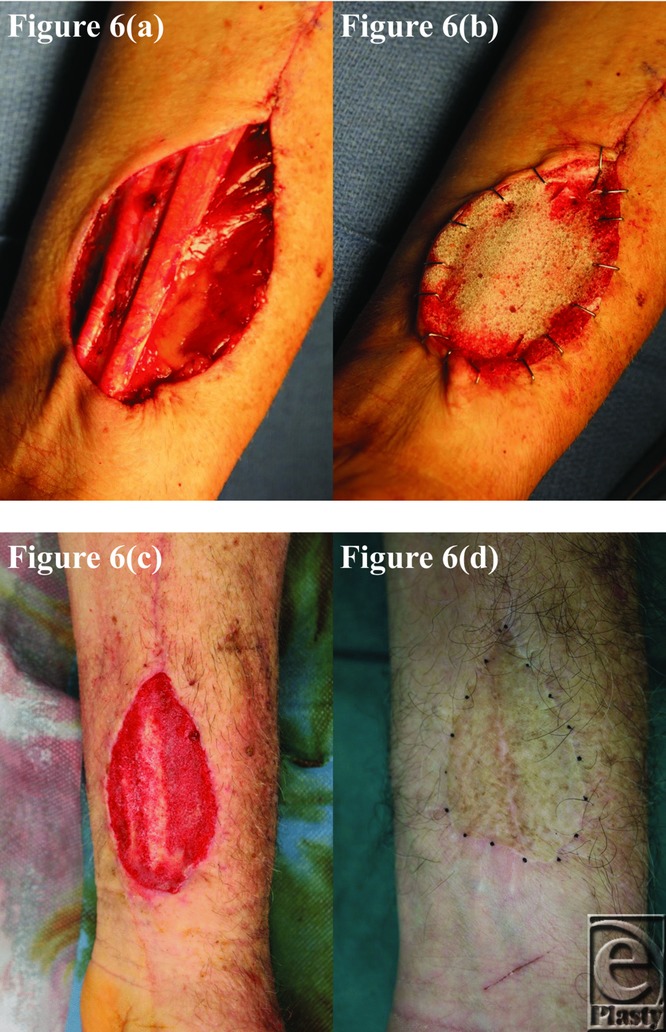
Patient 6 temporal series: (a) day 0 wound; (b) day 0 BTM implanted; (c) day 33 BTM delaminated and dermabraded; and (d) day 378 final graft BTM/scar. BTM indicates biodegradable temporizing matrix.

**Figure 7:**
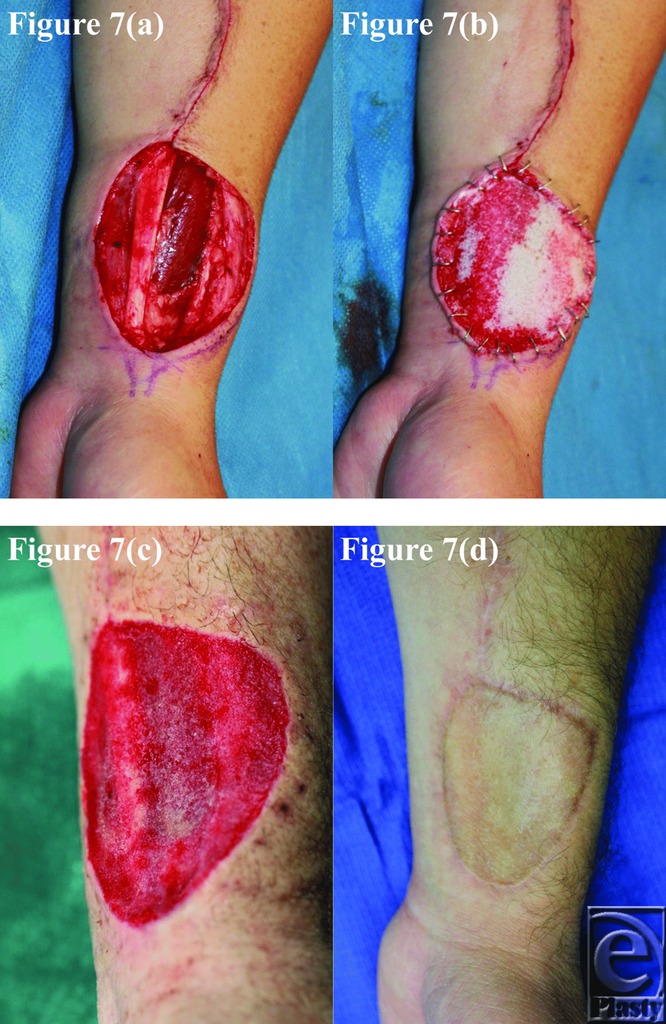
Patient 7 temporal series: (a) day 0 wound; (b) day 0 BTM implanted; (c) day 36 BTM delaminated and dermabraded; and (d) day 364 final graft BTM/scar.

**Figure 8:**
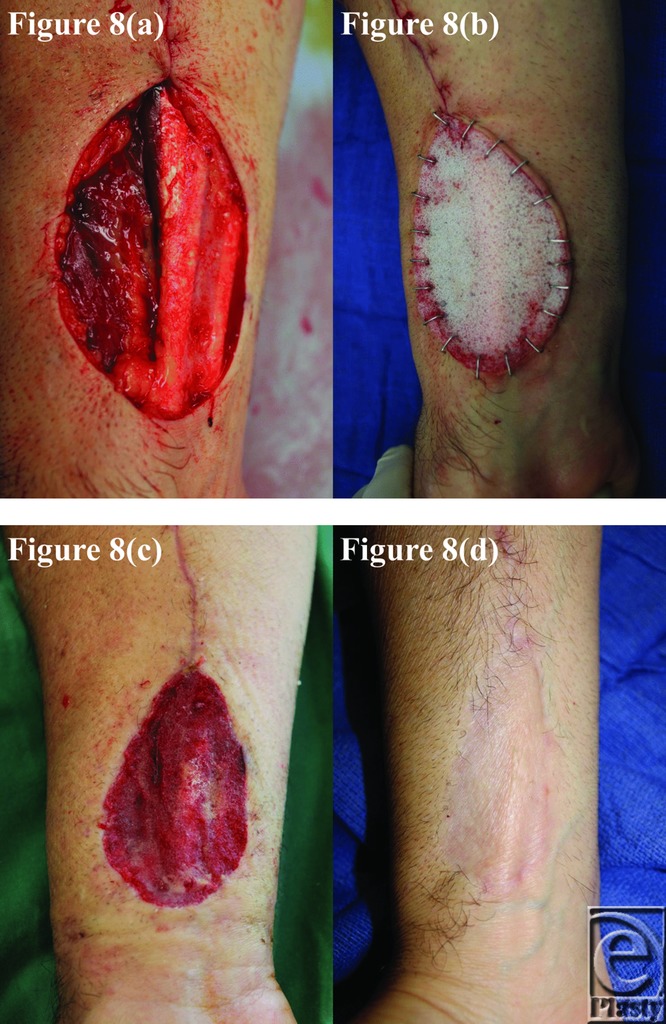
Patient 8 temporal series: (a) day 0 wound; (b) day 0 BTM implanted; (c) day 29 BTM delaminated and dermabraded; and (d) day 236 final graft BTM/scar. BTM indicates biodegradable temporizing matrix.

**Figure 9:**
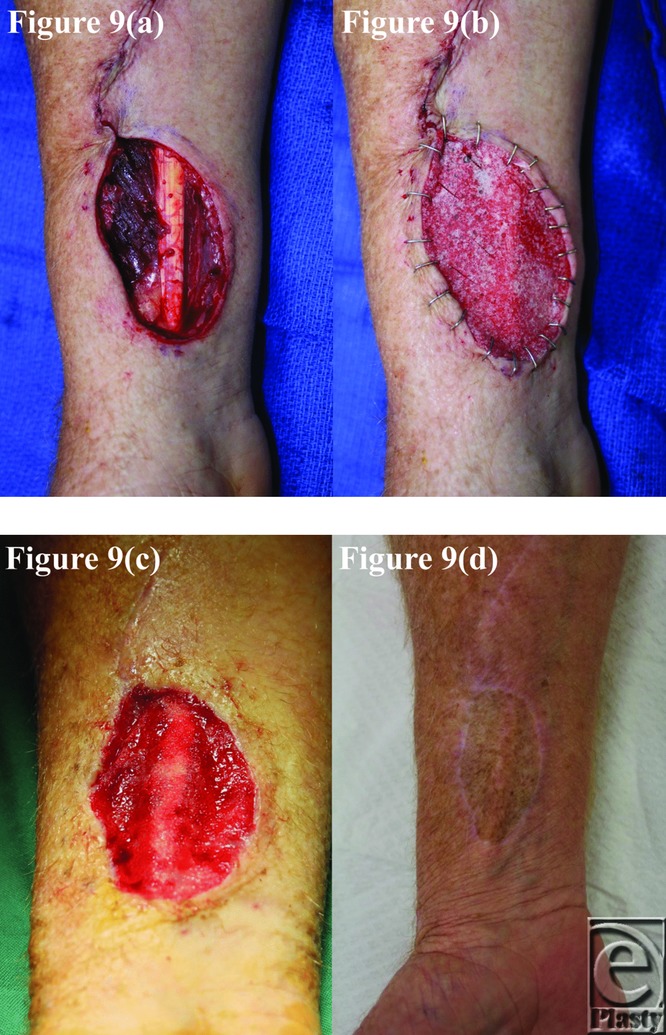
Patient 9 temporal series: (a) day 0 wound; (b) day 0 BTM implanted; (c) day 36 BTM delaminated and dermabraded; and (d) day 362 final graft BTM/scar. BTM indicates biodegradable temporizing matrix.

**Figure 10:**
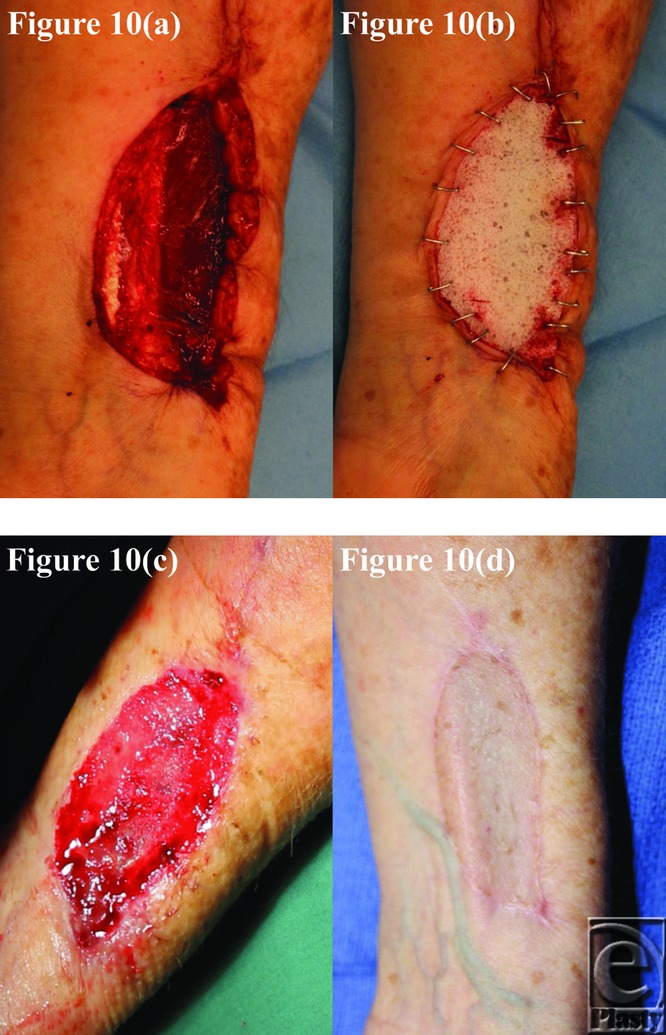
Patient 10 temporal series: (a) day 0 wound; (b) day 0 BTM implanted; (c) day 34 BTM delaminated and dermabraded; and (d) day 224 final graft BTM/scar. BTM indicates biodegradable temporizing matrix.

**Table 1:** Patient age, flap type, complications, BTM integration, day of grafting, graft take, and degree of wound contracture at the last reading (days post-implantation)

Patient	Age, y	Flap	Complications	% BTM integration	Graft day	% Wound area at grafting	% Take	% Initial wound area at last reading, (days)
1	63	FOC	Nil	100	34	103.03	100	102.36 (367)
2	62	RF	Nil	100	34	103.94	100	69.08 (424)
3	65	FOC	Nil	100	40	130.60	100	103.56 (391)
4	62	RF	Lost to follow-up after day 86	100	43	93.13	100	70.45 (86)
5	51	RF	Died of carotid blow-out after day 12					
6	61	RF	Nil	100	33	114.58	100	70.31 (378)
7	48	RF	Nil	100	36	100	100	73.10 (364)
8	50	RF	Died of local recurrence after day 236	100	29	88.15	100	61.67 (236)
9	55	RF	Nil	100	36	96.86	100	70.16 (362)
10	68	RF	Died of local recurrence after day 224	100	34	96.41	100	63.59 (224)

BTM indicates biodegradable temporizing matrix; FOC, fibular osseocutaneous, and RF, radial forearm.

**Table 2:** Mean wound areas, expressed as a percentage of the original areas of the wound prior to BTM implantation, of the first and second cohorts of free flap donor sites*

	First cohort (pilot)		Second cohort	
Donor Site	At time of SSG	At ≥1 y postimplantation	At time of SSG	At ≥1 y postimplantation
FOC flap	109.08 (3)	69.15 (3)	116.82 (2)	102.96 (2)
RF/UF flap	73.54 (4)	36.64 (4)	99.01 (7)	70.66 (4)

*Numbers of patients are given in brackets.

BTM indicates biodegradable temporizing matrix; SSG, split-skin graft; FOC, fibular osseocutaneous; RF, radial forearm; and UF, ulnar forearm.

**Table 3:** Scar assessment score summary (compared against the scores generated by the pilot study [first cohort])

	First cohort (pilot)	Second cohort
*MAPS*		
Average score	1.88 ± 1.25 (0–4)	1.33 ± 0.52 (1–2)
*POSAS*		
Observer scale		
Average score	2.63 ± 0.49	2.33 ± 0.47
Total score	15.75 ± 2.96 (12–20)	14.0 ± 2.83 (11–18)
Overall opinion	2.63 ± 0.74 (2–4)	2.33 ± 0.52 (2–3)
Patient scale		
Average score	1.88 ± 0.52	1.97 ± 0.78
Total score	11.29 ± 3.15 (6–16)	11.83 ± 4.67 (6–20)
Overall opinion	2.29 ± 1.38 (1–4)	1.83 ± 0.75 (1–3)

Results are presented as mean ± SD (range).

MAPS indicates Matching Assessment using Photographs with Scars; and POSAS, Patient and Observer Scar Assessment Scale.
